# Combined central and peripheral demyelination: a case report resembling encephalomyeloradiculoneuropathy

**DOI:** 10.3389/fneur.2023.1288546

**Published:** 2024-01-16

**Authors:** Xuan Zhou, Ali Peng, Chuan Li, Lin Li, Dan Yao, Yunfeng Hao, Chao Zhao, Qi Yan, Ying Li, Juntong Liu, Shuyu Liu, Wenping Zhu, Ying Du, Wei Zhang

**Affiliations:** ^1^Department of Neurology, Tangdu Hospital, Air Force Medical University, Xi’an, Shaanxi Province, China; ^2^Xi’an Medical University, Xi’an, Shaanxi Province, China

**Keywords:** combined central and peripheral demyelination, acute myelitis, Guillain-Barré syndrome, HEV infection, treatment

## Abstract

Combined central and peripheral demyelination (CCPD) is an extremely rare disease characterized by inflammatory demyelination in both the central and peripheral nervous systems. Herein, we reported case of a 14-year-old teenager who initially presented with the symptoms of acute myelitis (AM). Subsequently, the patient developed symptoms consistent with Guillain-Barré syndrome (GBS), which was supported by nerve conduction studies (NCV) and cerebrospinal fluid (CSF) analysis. Throughout the course of the disease, the patient experienced abdominal pain and abnormal liver function. After a comprehensive evaluation, we determined that the abnormal liver function was a result of hepatitis E virus (HEV) infection, which may have acted as a trigger for GBS. The patient was treated with corticosteroids, intravenous immunoglobulin and Rituximab, resulting in symptom relief and clinical improvement after therapy and follow-up. This case highlights the potential responsiveness and reversibility of CCPD. Given the heterogeneous nature of CCPD, there is currently no standardized diagnostic criteria or clear consensus on its treatment. Therefore, we recommend a thorough assessment of all possibilities and the development of consolidated management guidelines based on available data for this disorder.

## Introduction

Inflammatory demyelination diseases encompass a broad range of disorders characterized by demyelinating lesions mediated by inflammation. These disorders typically affect either the central nervous system (CNS) or peripheral nervous system (PNS). Combined central and peripheral demyelination (CCPD) is a rare condition that manifests as simultaneous or temporally separated demyelination in both the CNS and PNS. This particular disease is not commonly encountered in clinical practice (1). Diagnosing CCPD can be challenging due to the limited knowledge available, which is primarily derived from case reports and small case series (2–5). Moreover, some cases exhibited both CNS and PNS impairment were named as Encephalomyeloradiculoneuropathy (EMRN), which is similar with CCPD. Specific biomarkers were identified in CCPD. In 2013, Kawamura et al. found that anti-neurofascin-155 antibodies was positive in some CCPD patients (4). Recent studies reveal some antibodies were also associated with CCPD, such as anti-neurofascin186 antibody, antigalactocerebroside and antilactosylceramide (4, 6, 22, 23). While in EMRN, anti-neutral glycosphingolipid antibodies especially anti-lactosylceramide (LacCer) antibodies were identified (25). In this case report, we present a case report of a patient manifested the symptoms of acute myelitis (AM) and Guillain-Barré syndrome (GBS). Ultimately, she was diagnosed with CCPD.

## Case presentation

A 14-year-old girl with no significant medical or past history presented with weakness and tingling paresthesia in both lower extremities for 1 week. Additionally, she experienced stool and urinary retention. Prior to her presentation, she had transient abdominal pain but no fever or diarrhea. She had not received any vaccines within the past month. She was initially diagnosed with acute myelitis at a local hospital and was subsequently admitted to the neurology department of our hospital. The patient had no history of other neurological disorders, fever, weight loss, or night sweats. She also denied any changes in vision, difficulty swallowing or speaking, shortness of breath, chest pain, and loss of smell or taste sensation. Upon admission, the weakness in her lower extremities gradually worsened, while the stool and urinary retention improved. She required a wheelchair and was unable to stand up independently. A thorough examination was conducted. Initial findings and vital signs were within normal limits, and gastrointestinal, respiratory, and cardiovascular examinations were unremarkable. Neurological examination revealed that the patient was conscious, alert, and oriented. She had normal muscle bulk, but the strength of her bilateral lower extremities was graded as 2 on the muscle strength grading scale (with a maximum score of 5), with the right side being more affected than the left. Lower extremity reflexes and plantar reflex were absent, and there was hyperalgesia up to the bilateral groin.

The hemogram, liver, renal, and thyroid functions (TSH, Free T4), electrolytes (Na, K, Ca, P, Mg), muscle enzymes, vitamin B12 levels, as well as glycohemoglobin, were all within normal ranges. Virological tests (HIV, hepatitis B, hepatitis C, COVID-19) yielded negative results or fell within normal ranges. Immunological studies were conducted, and the results showed negative findings for antinuclear antibody, antiphospholipid IgM and IgG, anti-dsDNA, anticardiolipin IgM and IgG, anti-Sjogren antibody SSA and SSB, and extractable nuclear antigen. Visual-evoked potentials (VEPs) demonstrated normal P100 latencies bilaterally (right: 106 ms, left: 108 ms). SEP also exhibited normal latencies bilaterally. The CSF examination revealed normal intracranial pressure, a slightly elevated white blood cell count (20*10^6/L), and an elevated protein level (780 mg/L, normal range: 150–450 mg/L). CSF culture yielded negative results, and CSF cytology showed no malignant cells. Cerebrospinal fluid analysis indicated a normal albumin level (536 mg/L), normal IgG level (75.2 mg/L), and a normal CSF-to-serum albumin ratio (12.3). Serum albumin and serum IgG levels were also within normal ranges. Aquaporin-4, anti-MBP antibodies, and anti-MOG antibodies were not detected. CSF and blood oligoclonal bands were absent.

**Figure 1:**
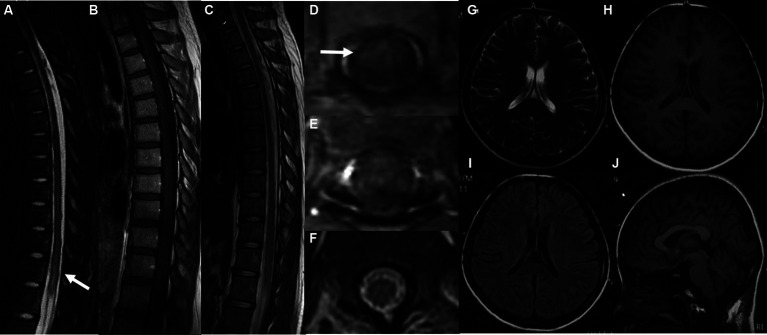
MRI images of the spinal cord both before and after therapy **(A,C,D,F)**. Gadolinium-enhanced axial T1-weighted MRI of the spinal cord **(B,E)**. MRI of the brain **(G–J)** revealed the absence of brain lesions. The sagittal T1-weighted image **(A)** and axial T1-weighted image **(D)** of the thoracic spinal cord before therapy revealed an abnormal signal in the thoracic and lumbar spinal cord (T11-L1). We did not observe obvious enhancement of spinal lesion in enhanced T1-weighted MRI **(B,E)**. After therapy, the sagittal T1-weighted image **(C)** and axial T1-weighted image **(F)** demonstrated a significant reduction in the size of the previously observed lesions.

In the imaging assessments, Magnetic Resonance Imaging (MRI) revealed the absence of brain lesions (Figures 1G–J), while spinal cord resonance imaging and gadolinium-enhanced MRI of the spinal cord demonstrated mild cord edema, but we did not observe obvious enhancement of spinal lesion in enhanced T1-weighted MRI (Figures 1A,B,D,E). The spinal cord MRI did not indicate any manifestations of acute disseminated encephalomyelitis (ADEM) or multiple sclerosis (MS). In the early stages of treatment, the patient received methylprednisolone therapy (1,000 mg once daily) for a duration of 3 days, followed by a gradual reduction of the methylprednisolone dosage over the course of 1 week. Subsequent to the treatment, some improvement in muscle strength was observed (lower limbs: MRC grade: 2).

**Figure 2:**
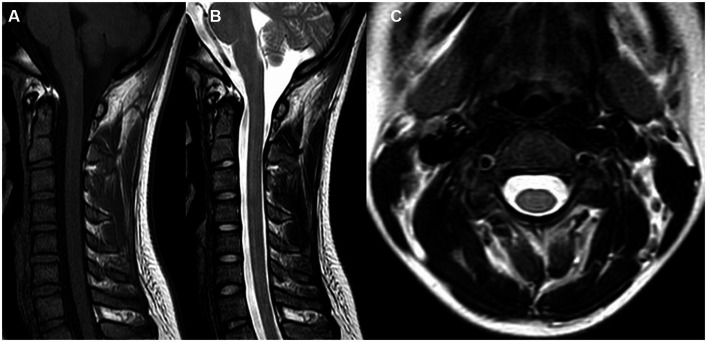
MRI images of the cervical spinal cord **(A–C)** were obtained. These included sagittal T1-weighted **(A)**, T2-weighted **(B)**, and axial T1-weighted **(C)** images. Notably, no lesions were observed in the cervical spinal cord.

However, during the course of treatment, the patient experienced abdominal pain and exhibited abnormal liver function, as indicated by elevated alanine aminotransferase (ALT) levels of 205 U/L and aspartate aminotransferase (AST) levels of 60 U/L. Notably, muscle enzyme levels remained within the normal range. Screening for Epstein–Barr Virus, Cytomegalovirus, Herpes Simplex Virus, and Herpes Zoster Virus yielded negative results. Even after receiving hepatoprotective therapy, the patient’s ALT and AST levels did not return to normal. Additionally, the patient developed acute weakness in the upper limbs, which rapidly progressed to tetraplegia (bilateral lower limbs: MRC grade 2, left upper limb: MRC grade 2, and right upper limb: MRC grade 3). The patient’s deep tendon reflexes remained hyporeflexic. MRI of the spinal cord revealed no lesions in the cervical spinal cord (Figure 2). Subsequently, the patient underwent a series of electromyograms (EMGs) and nerve conduction studies (NCVs). NCVs revealed peripheral neuropathy (Table 1). CSF analysis showed protein cytological dissociation, with an increased protein level of 1317.5 mg/L and a normal cell count of 4 cells/mm^3^. The patient was negative for anti-glycolipid (GM1, GM2, GM3, GD1a, GD1b, GD3, GT1b, GQ1b, galactocerebroside). No oligoclonal bands were detected. The patient displayed the classic triad of weakness of extremities, albuminocytologic dissociation was observed in the CSF sample, which strongly supported the diagnosis of Guillain-Barré Syndrome (GBS). In light of the abnormal liver function, a comprehensive examination was conducted, ultimately determining that the patient’s abnormal liver function was a result of HEV infection. Intravenous immunoglobulin (IVIg) was administered at a dose of 0.4 g/kg/day and continued for 5 days. The patient demonstrated some improvement after completing the course of IVIg. She regained full strength (5/5) in all muscle groups in the upper extremities and had a strength of 3/5 in the lower extremities. As liver function continued to improve, the patient received Rituximab (RTX) infusion at a dose of 100 mg, with one-week intervals for two times. At four-month follow-up, her neurological condition is stable. She experienced progressive improvement in muscle strength in all limbs and was able to briefly stand with assistance. Follow-up MRI of the spinal cord revealed evidence of lesion improvement (Figures 1C,F). NCVs indicated improvement in demyelinating polyneuropathy (Table 2).

**Table 1:** A summary of nerve conduction studies demonstrating demyelinating polyneuropathy.

Motor NCS					
Nerve	Site		Latency (ms)	Amplitude (mV)	Conduction velocity (m/s)
Peroneal	Right	Ankle	2.9	2.6	-
		Fibular head	9.2	2.3	48
		Popliteal	10.7	2.3	47
	Left	Ankle	3.1	3.4	-
		Fibular head	8.9	3.0	53
		Popliteal	10.4	2.9	47
Tibial	Right	Ankle	4.2	13.8	-
		Popliteal	10.8	9.8	55
	Left	Ankle	4.1	15.0	-
		Popliteal	11.2	11.8	52
Median	Right	Wrist	2.9	5.9	-
		Elbow	6.2	5.8	64
	Left	Wrist	2.8	5.8	-
		Elbow	6.2	5.6	60
Ulnar	Right	Wrist	2.4	8.2	-
		Elbow	5.5	8.1	61
		Above elbow	7.3	7.6	56
	Left	Wrist	2.4	6.3	-
		Elbow	5.3	5.9	62
		Above elbow	7.0	5.9	59
Radial	Right	Extensor indicis proprius	1.7	2.3	-
		Forearm	3.3	2.2	63
		Above elbow	4.8	2.2	67
	Left	Extensor indicis proprius	2.0	2.4	-
		Forearm	3.5	2.7	67
		Above elbow	4.9	2.4	71
Femoral	Right	Vastus medialis	5.9	0.6	-
	Left	Vastus medialis	6.4	0.7	-
Sensory NCS					
Nerve		Peak Latency (ms)	Amplitude (uV)	Conduction velocity (m/s)	
Ulnar	Right	1.7	61.5	59	
	Left	1.7	69.4	59	
Superficial	Right	1.9	16.6	58	
	Left	2.1	14.4	52	
Late responses					
Nerve		Latency (ms)			
R Tibial F-wave		49.9			
L Tibial F-wave		47.1			
R Median F-wave		23.8			
L Median F-wave		23.4			
R Ulnar F-wave		29.9			
L Ulnar F-wave		29.9			

**Table 2:** A summary of nerve conduction studies at following-up.

Motor NCS					
Nerve	Site		Latency (ms)	Amplitude (mV)	Conduction velocity (m/s)
Peroneal	Right	Ankle	3.2	1.1	-
		Fibular head	8.9	1.0	53
		Popliteal	10.0	0.9	55
	Left	Ankle	3.8	0.7	-
		Fibular head	9.4	0.5	54
		Popliteal	10.6	0.5	50
Tibial	Right	Ankle	4.2	6.1	-
		Popliteal	10.8	4.6	55
	Left	Ankle	4.4	9.2	-
		Popliteal	11.3	5.9	53
Median	Right	Wrist	2.8	8.1	-
		Elbow	6.1	8.0	62
	Left	Wrist	2.7	8.7	-
		Elbow	5.9	8.8	64
Ulnar	Right	Wrist	2.2	8.0	-
		Elbow	5.1	8.2	64
		Above elbow	6.7	8.1	63
	Left	Wrist	2.2	7.3	-
		Elbow	5.1	7.2	64
		Above elbow	6.7	6.9	63
Sensory NCS					
Nerve		Peak Latency (ms)	Amplitude (mV)	Conduction velocity (m/s)	
Nerve		Peak Latency (ms)	Amplitude (mV)	Conduction velocity (m/s)	
Ulnar	Right	1.7	80.5	57	
	Left	1.8	73.8	55	
Superficial	Right	1.8	17.4	61	
Late responses					
Nerve		Latency (ms)			
R Tibial F-wave		42.8			
L Tibial F-wave		43.2			
R Median F-wave		22.6			
L Median F-wave		23.2			

## Discussion

In this report, we present a case of CCPD in a teenage patient. Our case is notable both because the presence of central and peripheral demyelinating processes in one patient is a rare phenomenon. CCPD is characterized by demyelination affecting both the central and peripheral nervous systems. It remains unclear whether CCPD represents a distinct disorder separate from classical demyelinating diseases (6). Currently, there are no standardized criteria for diagnosing CCPD. However, a previous nationwide survey conducted in Japan established criteria for CCPD, which encompassed a diverse range of clinical manifestations involving both the CNS and PNS, such as encephalopathy, cranial neuropathy, myeloradiculoneuropathy, or Guillain-Barré syndrome (6). The synchronous occurrence of GBS and AM in CCPD appears to be rare and variable. Early-stage diagnosis of CCPD, particularly when symptoms involving both the CNS and PNS are present simultaneously, poses challenges. Published literature on cases reporting such concurrent conditions is limited. The underlying pathophysiological mechanism of CCPD remains elusive. It is uncertain whether the same antigenic target exists in both the PNS and CNS, or if there is a cross-reactive immune response to insults affecting either system (3). The immune response elicited by antecedent infections may cross-react with components of the peripheral nerves due to the sharing of common epitopes (7). Overall, CCPD encompasses a spectrum of conditions, including acute, relapsing, and chronic subtypes (4).

Anti-NF155 antibodies is a protein expressed on both central and peripheral myelin. It was first reported in CCPD cases in 2013 (4). Previous study has reported the high prevalence of anti-NF155 antibodies in CCPD (45.5–86%) (4). However, the positivity rates for anti-NF155 antibodies in CCPD differ among studies and ethnicities, some studies revealed the absent of antibody in this disorder. Therefore, we cannot exclude that anti-NF155 antibodies may be present in particular subgroups of patients with CCPD. So far, we are unable to demonstrate a pathophysiologic link between this case of CCPD and anti-NF155 antibody.

The manifestation of CCPD is similar with EMRN, but their pathogenesis remains to be fully elucidated. Although CCPD and EMRN both exhibit CNS and PNS impairments, the detailed pathogenesis of both disorders is still unclear at present. The clinical features of CCPD include a chronic onset, albumin-cytologic dissociation of cerebrospinal fluids, and a low frequency of oligoclonal IgG bands (OCB) positivity. In contrast, EMRN is an acute or subacute progressive disease that causes motor weakness, myelopathy, neuropathy, encephalopathy, and dysautonomia and mild CSF pleocytosis. CCPD is characterized by multifocal acquired inflammatory demyelinating sensory and motor neuropathy. While in EMRN, peripheral neuropathy is axonal, with or without demyelinating neuropathy (23, 24). Some autoantibodies, such as anti-lactosylceramide antibody, which was identified in CCPD was also found in EMRN. However, the specific biomarkers were identified for both disorders: anti-NF155 for CCPD and anti-neutral glycolipid (especially anti-lactosylceramide) for EMRN (6, 24, 27, 28). Our case exhibited some common features of EMRN such as acute motor weakness and dysautonomia, which are not common in CCPD. However, the albumin-cytological dissociation of cerebrospinal fluids and absent of OCB positivity, which is fulfilling the main features of CCPD. Ultimately, we diagnosed the case CCPD based on criteria of Ogata et al. (6). The clinical course of this case highlights the complexity of CCPD. Accurate diagnosis is crucial to ensure the patient receives the most appropriate and effective e treatment. Nerve conduction studies and spinal cord MRI are valuable diagnostic tools. Compared with previous patients with EMRN, our case had a progressing clinical course (25).

Furthermore, the patient presented the symptoms of GBS and we finally revealed HEV infection might be a trigger of GBS. GBS is a heterogeneous disease characterized by acute immune-mediated polyneuropathies, often associated with preceding viral or bacterial infections (7, 8). Previous studies have indicated the most frequently identified infectious triggers for GBS include *Campylobacter jejuni* infection, influenza-like illnesses, cytomegalovirus, Epstein–Barr virus (EBV), and varicella-zoster virus. Additionally, hepatotropic viruses such as hepatitis A, hepatitis B, and hepatitis C have been implicated as triggering agents (9). Recently, several case reports and studies have reported an association between HEV infection and GBS (10–13). It has been suggested that HEV infection may be responsible for 5 to 11% of sporadic GBS cases in developed countries (14). A study conducted in Bangladesh further confirmed the link between HEV and GBS (15). The exact mechanism underlying GBS associated with HEV infection remains uncertain. One possibility is that HEV directly infects the peripheral nerve roots or the central nervous system, causing neural cell injury, as demonstrated by its ability to replicate in various human neuronal-derived cell lines (16). Another potential mechanism involves molecular mimicry, in which the virus indirectly triggers the disorder by sharing similar antigenic characteristics with components of the peripheral nerves (7). In our patient, who presented with symptoms of GBS and abnormal liver function, we suspected that HEV infection may have triggered the development of GBS based on previous studies (15). Notably, elevated liver enzymes, including alanine or aspartate aminotransferase, are commonly observed in GBS patients with HEV infection, occurring in approximately 75% of cases according to combined series data (17, 18). Confirmation of HEV infection can be established through etiology testing, specifically when serum anti-HEV IgG is positive. As a result, we recommend considering HEV testing in GBS patients.

Due to its rarity, the treatment of CCPD has not been thoroughly investigated. In our case, the patient underwent steroid therapy along with additional administration of IVIg therapy, resulting in a transient response (19). Subsequently, the patient received RTX and exhibited a favorable response. Although similar cases have been reported in previous literature, the optimal therapy for CCPD remains uncertain. Steroids, IVIg, and plasmapheresis have shown efficacy in treating CCPD. Acute or subacute combined CCPD has rarely been observed in adults, with documented successful treatment using plasmapheresis and IV IgG or IV corticosteroids. More recently, Rituximab has also demonstrated positive outcomes in patients with CCPD (1, 20). In another case, RTX was utilized as a third-line therapy following treatment with steroids, IVIg, and Natalizumab, resulting in clinical and radiological improvement without relapses during the 28-month follow-up period (21). Moreover, in a placebo-controlled randomized controlled trial of relapsing–remitting MS (RRMS) patients, RTX was found to drastically decrease the counts of contrast-enhancing lesions as well as volumes of T2 lesions. Furthermore, it reduced the proportion of patients with relapse at 48 weeks (RTX:20.3% vs. placebo: 40.0%) (26). Therefore, aggressive immunomodulation therapy should be considered to induce remission in patients who do not respond to these treatments.

In conclusion, the clinical presentation of CCPD exhibits significant heterogeneity, making the diagnosis challenging in clinical practice. Therefore, it is imperative to establish definitive international criteria for CCPD in larger sample sizes and across multiple centers. We recommend the utilization of MRI and electrophysiological examinations for patients suspected of having CCPD. In cases where both AM and GBS manifestations are observed, we suggest conducting anti-AQP4, MOG, and NCV assessments to achieve a precise diagnosis. Furthermore, it is crucial to exclude HEV infection in patients presenting with GBS symptoms and abnormal liver function. If patients exhibit a poor response to steroids and intravenous immunoglobulin, RTX should be considered as a secondary treatment option.

## Data availability statement

The raw data supporting the conclusions of this article will be made available by the authors, without undue reservation.

## Ethics statement

Written informed consent was obtained from the individual(s), and minor(s)’ legal guardian/next of kin, for the publication of any potentially identifiable images or data included in this article.

## Author contributions

XZ: Writing – review & editing, Writing – original draft. AP: Writing – original draft, Writing – review & editing. CL: Writing – original draft, Writing – review & editing. LL: Writing – review & editing. DY: Writing – review & editing. YH: Writing – review & editing. CZ: Writing – review & editing. QY: Writing – review & editing. YL: Writing – review & editing. JL: Writing – review & editing. SL: Writing – review & editing. WNZ: Supervision, Writing – review & editing. YD: Writing – review & editing. WIZ: Writing – review & editing, Supervision.
